# Whole genome re-sequencing to identify suppressor mutations of mutant and foreign *Escherichia coli* FtsZ

**DOI:** 10.1371/journal.pone.0176643

**Published:** 2017-04-26

**Authors:** Kiani A. J. Arkus Gardner, Masaki Osawa, Harold P. Erickson

**Affiliations:** Department of Cell Biology, Duke University Medical Center, Durham, North Carolina, United States of America; University of Iowa, UNITED STATES

## Abstract

FtsZ is an essential protein for bacterial cell division, where it forms the cytoskeletal scaffold and may generate the constriction force. We have found previously that some mutant and foreign FtsZ that do not complement an *ftsZ* null can function for cell division in *E*. *coli* upon acquisition of a suppressor mutation somewhere in the genome. We have now identified, via whole genome re-sequencing, single nucleotide polymorphisms in 11 different suppressor strains. Most of the mutations are in genes of various metabolic pathways, which may modulate cell division indirectly. Mutations in three genes, *ispA*, *accD* and *nlpI*, may be more directly involved in cell division. In addition to the genomic suppressor mutations, we identified intragenic suppressors of three FtsZ point mutants (R174A, E250K and L272V).

## Introduction

FtsZ is an essential bacterial protein that assembles into the Z ring at midcell. FtsZ provides the cytoskeletal framework for the cell division machinery, which includes a number of downstream and interacting proteins required for division. FtsA and ZipA are membrane associated proteins that bind the highly conserved C-terminal 15- to 17-amino acids of FtsZ, thereby tethering FtsZ to the membrane [1–4]. In *Escherichia coli*, upon Z-ring assembly and binding to ZipA or FtsA, FtsK is the first downstream protein to be recruited, and the others follow in a mostly linear fashion [5–7]. In vitro experiments have shown that FtsZ can assemble Z rings in liposomes, and these reconstituted Z rings can generate a constriction force. Both assembly and constriction occur without the need for any other proteins [8]. When combined with FtsA, FtsZ can achieve division of liposomes [9]. The downstream proteins are mostly involved with remodeling the peptidoglycan wall in response to the localization and initial constriction by FtsZ. The overall process of cell division has been reviewed by Adams and Errington [10] and Lutkenhaus et al [11]; more focused reviews of FtsZ are by Erickson et al [12] and Mingorance et al [13].

Previous work [14] has tested whether FtsZ from divergent foreign bacteria (*Bacillus subtilis* and *Mycoplasma pulmonis*) can function for *E*. *coli* division. The sequences of these are only ~50% identical to that of *E*. *coli* FtsZ, and we expected them to be non-functional if they needed to associate with any *E*. *coli* proteins. However, we found that they did function given two conditions. First, the foreign FtsZ must be supplied with the conserved C-terminal peptide from *E*. *coli*, which binds *E*. *coli* FtsA and ZipA. Second, a suppressor mutation in the *E*. *coli* genome must be obtained. A number of suppressor strains were obtained in that study, each presumed to contain a mutation somewhere in the *E*. *coli* genome that allowed the partially deficient foreign FtsZ to achieve cell division. Suppressor strains were also generated that facilitated function of various point mutations in FtsZ and of a C-terminal fusion of YFP. Suppressor strains were partially cross-reactive. For example, a suppressor strain generated by *M*. *pulmonis* FtsZ also suppressed three *E*. *coli* mutants with short or long inserts, as well as the point mutant Q47K [14].

How does a genomic mutation facilitate cell division by the foreign or mutant FtsZ? One possibility is that these suppressor mutations were loss-of-function mutations in negative regulators of FtsZ, or in pathways affecting the integrity of the cell wall, thus allowing for function of partially defective FtsZ. Another possibility is that multiple pathways of basic and secondary metabolism feed back into cell division, and mutations at various points in these pathways might facilitate division by a partially defective FtsZ. Our present study favors this latter scenario.

Our several attempts to identify these genomic mutations by traditional methods of bacterial genetics were unsuccessful, so we turned to whole genome re-sequencing technologies [15, 16]. Hayashi et al [17] sequenced the entire genomes of two K-12 *E*. *coli* strains (W3110 and MG1655) and showed that the mutation frequency between these strains was very modest, with only eight base pair changes. The strain we used to generate the suppressors, designated JKD7-1, is a W3110 strain [18], but was separated at an unknown time from the stock strain sequenced by Hayashi et al. In addition to identifying the suppressor mutation, our re-sequencing project has identified the number and type of mutations between our lab strain and the published W3110 sequence.

In the present study, we have sequenced the genomes of some of the previously reported suppressor strains [14], as well as some newly generated ones. We expected each of these strains to have a single mutation somewhere in the genome that was responsible for the suppressor phenotype. In 11 strains we have successfully identified a single genomic mutation that is likely responsible for the suppressor phenotype. We also identified intragenic suppressors of three FtsZ mutants that were so deleterious that they neither complemented nor generated suppressor strains.

## Materials and methods

### Bacterial strains and growth conditions

*E*. *coli* strain JKD7-1/pKD3 [18] was used for complementation assays and for generating suppressor strains. JKD7-1 has a chromosomal *ftsZ* null mutation (caused by an inserted kanamycin cassette) and is supported by a pKD3 rescue plasmid, which expresses wt FtsZ and is temperature sensitive for replication. At 42°C, pKD3 replication is restricted and wt FtsZ is eliminated. The cell strain is maintained at 30°C in Repression Media: Luria-Bertani (LB) media containing 100 μg/mL ampicillin (selecting for the pKD3 plasmid), 100 μg/mL of kanamycin (selecting for the inserted kanamycin cassette), 34 μg/mL of chloramphenicol (selecting for the mutant or foreign *ftsZ* on the pJSB2 plasmid [19]), and 0.2% (w/v) glucose (suppressing expression of the pJSB2-FtsZ protein).

### Plasmid construction and suppressor strain generation

pJSB2 was used to express the mutant or foreign FtsZ as previously described [14, 19]. pJSB2 was derived from the pBAD plasmid, which has an arabinose-inducible promoter. Foreign and mutant *ftsZ* was cloned into the pJSB2. The yellow fluorescent protein (YFP) was the Venus variant [20], which we have found to be superior to other green fluorescent proteins for fusion to FtsZ (Venus gave a functional insert where eGFP was non-functional [21]). The foreign *ftsZ* were from *Bacillus subtilis*, *Azotobacter vinelandii*, *Mycoplasma pulmonis* and *Pseudomonas aeruginosa*. Chimeric molecules with the *E*. *coli* FtsZ C-terminal tail (aa 325–383) fused to the globular domain of FtsZ from foreign bacteria were spliced with a silent SpeI site introduced at aa 326 in EcFtsZ in pJSB2 [14].

JKD7-1/pKD3 cells containing pJSB2 mutant/foreign *ftsZ* were grown in Repression Media at 30°C overnight. The cultures were then diluted 1:1000, and 10^6^ cells were plated on agar containing Induction Media: LB media containing 100 μg/mL kanamycin, 34 μg/mL chloramphenicol and 0.2% (w/v) arabinose (to induce expression of the mutant FtsZ protein from pJSB). Plates were grown at 42°C to eliminate the temperature sensitive pKD3 rescue plasmid. Suppressor mutations that permitted the mutant *ftsZ* to function for division arose at a frequency of 10^−4^ to 10^−6^. These were verified to be true genomic suppressors as previously described [14].

### Genome sequencing and analysis

Genomic DNA was isolated from stationary phase liquid cultures of suppressor strains by a standard alkaline lysis DNA extraction protocol. Samples were prepared for sequencing using the Illumina Genomic DNA Sample Prep Kit (San Diego, CA). Briefly, DNA was sheared to 200–800 bp using a nebulizer. 3’ A-overhangs were added to the blunt-ended fragments, and the library was amplified with 18 rounds of PCR. The resultant libraries were sequenced using the Illumina Genome Analyzer II platform, with data outputs of 36 nucleotide single-end reads. Genome sequencing was performed by the Duke University Institute for Genome Sciences and Policy Genome Sequencing and Analysis Core, and by the Georgia Health Sciences University Integrated Genomics Core. Data sets were aligned to the annotated *E*. *coli* W3110 genome ([17] Genbank accession number AP009048) using the Bowtie sequence alignment package (http://bowtie-bio.sourceforge.net) and Samtools utilities (http://samtools.sourceforge.net). Single nucleotide mutations were identified manually in the Integrated Genome Viewer (http://www.broadinstitute.org/software/igv). Each mutation identified by the genomic sequence analysis was confirmed by PCR and Sanger sequencing of the relevant locus.

### Complementation in slow growth conditions

JKD7-1/pKD3 cells containing pJSB2 expressing a mutant or foreign FtsZ were grown in Repression Media at 30°C overnight. The cultures were then diluted 1:1000, and 10^6^ cells were plated on dishes containing Induction Minimal Media: M9 minimal media-agar containing 100 μg/mL kanamycin, 34 μg/mL chloramphenicol and 0.2% (w/v) arabinose. An equal volume of cells was also plated on Repression Minimal Media plates and grown at 30°C. A variant form of FtsZ was considered to complement if it produced a lawn of cells equivalent to that on the Repression plate at the permissive temperature. Constructs that did not complement produced no colonies, suggesting that growth on Repression Minimal Media is not favorable to generation of suppressor strains.

### Search for intragenic suppressors

Libraries of random mutants were made with the GeneMorph II Random Mutagenesis Kit from Agilent Technologies (Santa Clara, CA). The mutant *ftsZ* gene of interest (R174A, E250K or L272V) in pJSB2 was amplified by PCR using the primers 5’ccgccattcagagaagaaaccaattgtcca3’ and 5’ttgatgcctggcagttccctactctcgcat3’, which amplify the entire *ftsZ* with an extra 200 bp of pJSB2 sequence flanking each end. The PCR used the Mutazyme II DNA polymerase, which randomly misincorporates nucleotides during DNA synthesis. Protocol instructions were followed to achieve a predicted mutational frequency of 1–2 nucleotide changes in each gene. Mutant library PCR products were cloned into pJSB2 using BamHI and HindIII restriction sites flanking the *ftsZ* gene to produce a library of pJSB-mutFtsZ*.

The pJSB-mutFtsZ* library was transformed into JKD7-1/pKD3 and screened for complementation, as described above. Colonies that grew on Induction plates at 42°C were selected as potential second-site suppressors. Plasmids were isolated and transformed into fresh DH5α to ensure that DNA sent for sequencing did not contain any residual pKD3 rescue plasmid. Plasmids that showed a second site mutation were retransformed into JKD7/pKD3 to confirm the rescue. The new mutation was also created as a single mutation in pJSB2, and tested for complementation.

## Results

### Generation of suppressor strains

**Fig 1:**
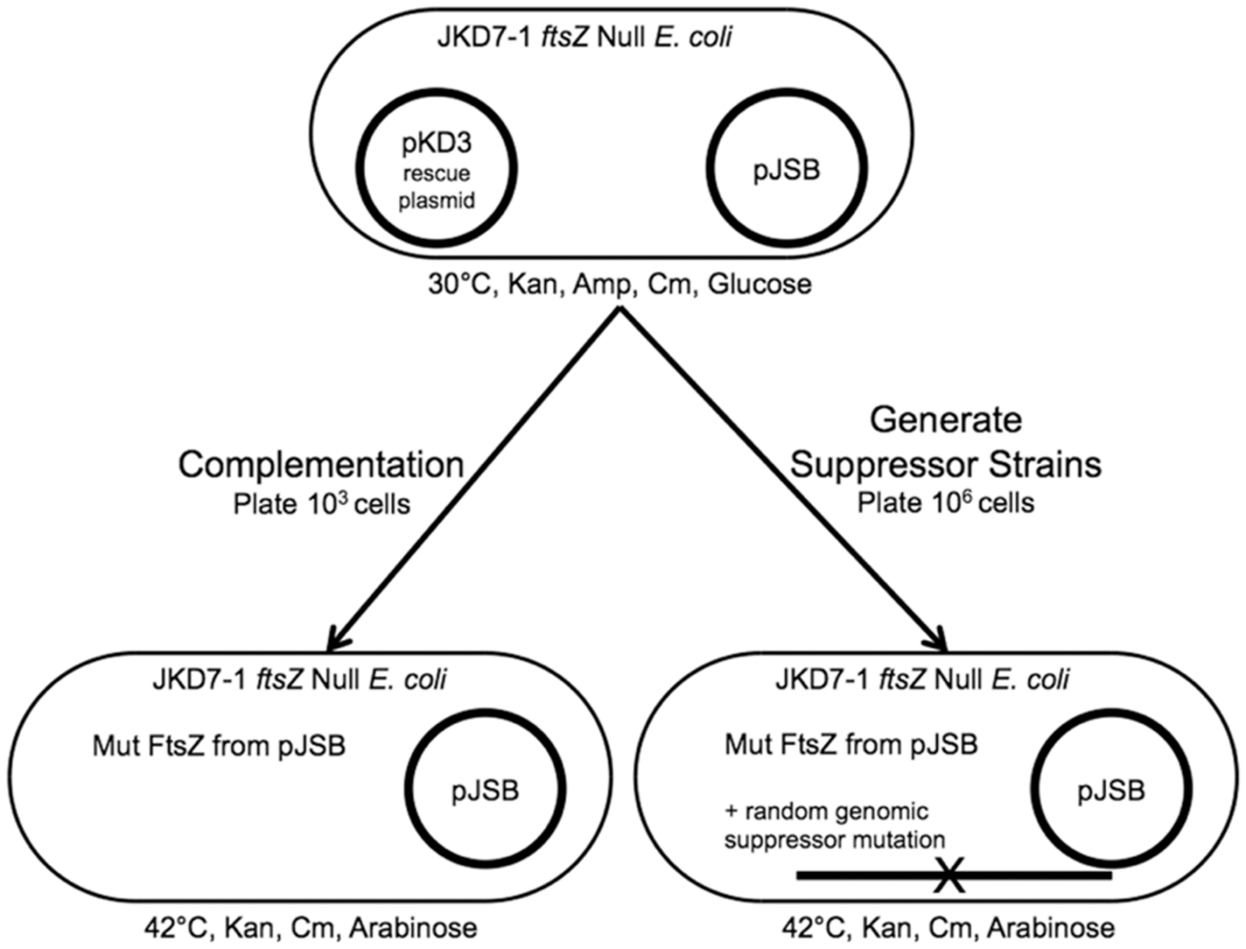
A schematic of the assay used to test a foreign or mutant FtsZ for complementation of the *ftsZ*-null phenotype or the generation of a suppressor strain, as described in results and previously [14].

Suppressor strains were generated using a modification of the complementation assay originally developed by Dai and Lutkenhaus, and subsequently by Osawa and Erickson [14, 18] (Fig 1). JKD7-1 cells, which have a chromosomal *ftsZ* null mutation (*ftsZ*::*kan*), are supported by a pKD3 rescue plasmid that expresses wild type FtsZ and is temperature sensitive for replication. The foreign or mutant FtsZ to be tested is produced by the arabinose-inducible plasmid, pJSB2 [19, 22]. In the presence of arabinose (Induction Media) and after several generations at the restrictive temperature of 42°C, wt FtsZ is depleted and the pJSB plasmid becomes the only source of FtsZ available for cell division. The ability of the mutant FtsZ to function for cell division is demonstrated by the ability of plated cells to form colonies. If the mutant FtsZ protein is fully functional for cell division, the number of colonies on the Induction plate at the restrictive temperature (cells expressing FtsZ solely from pJSB) was at least 80% of the total cells plated (10^3^ cells plated, as measured on the Repression plate at the permissive temperature). If the FtsZ could not complement the *ftsZ*-null phenotype, we plated a larger number of cells to identify any suppressor colonies. When 10^6^ cells were plated on Induction Media at 42°C, some smaller colonies were observed at a frequency usually between 10^−4^ and 10^−5^, and as low as 10^−6^ in the case of FtsZ-D212G. These colonies were ampicillin sensitive and dependent on arabinose for growth, indicating that the rescue plasmid was eliminated and the cells were dependent on the pJSB2-FtsZ. These colonies were identified as suppressor strains, in which a randomly arising genomic mutation allowed a non-complementing FtsZ protein to function for cell division. Further testing [14] confirmed that the mutation was genomic, rather than on the pJSB2 plasmid.

The FtsZ that generated each suppressor strain is noted in the strain names. For example, we the abbreviation SUP[Av] refers to suppressor strains generated using the pJSB-Av plasmid expressing FtsZ from *Azotobacter vinelandii*. In the instances where two or more suppressor strains were characterized from a single pJSB construct, we have designated these by appending a number, e.g., SUP[Av3] and SUP[Av4]. All abbreviations are explained in Table 1.

**Table 1:** Suppressor strains are designated in column 1 as in [14], and are described in more detail in column 2.

Strain	Description	
SUP[Av3]	Third strain generated against *A*. *vinelandii* FtsZ	
SUP[Av4]	Fourth strain generated against *A*. *vinelandii* FtsZ	This study
SUP[Bs/EcCt1]	First strain generated against *B*. *subtilis* FtsZ with the *E*. *coli* FtsA-binding peptide	This study
SUP[Bs/EcCt2]	Second strain generated against *B*. *subtilis* FtsZ with the *E*. *coli* FtsA-binding peptide	This study
SUP[D212G3]	Third strain generated against D212G FtsZ	[14]
SUP[D212G9]	Ninth strain generated against D212G FtsZ	This study
SUP[Mpu/EcCt13]	13^th^ strain generated against the *M*. *pulmonis* FtsZ with the *E*. *coli* FtsA-binding peptide	[14]
SUP[Mpu/EcCt28]	28^th^ strain generated against the *M*. *pulmonis* FtsZ with the *E*. *coli* FtsA-binding peptide	[14]
SUP[Q47K2]	Second strain generated against Q47K FtsZ	[14]
SUP[CtYFP1//Q47K]	Double suppressor, generated first against CtYFP FtsZ, then against Q47K FtsZ	[14]
SUP[CtYFP1//D96A]	Double suppressor, generated first against CtYFP FtsZ, then against D96A FtsZ	[14]
SUP[CtYFP1//D212G-YFP]	Double suppressor, generated first against CtYFP FtsZ, then against D212G-YFP FtsZ	This study
SUP[CtYFP2]	Second strain generated against CtYFP FtsZ	[14]

In three cases we generated a double-suppressor strain. These were first generated as a suppressor strain for the FtsZ-CtYFP, an FtsZ with a C-terminal YFP fusion. That strain was then transformed again with an FtsZ point mutant, which required selection of a second suppressing genomic mutation. These strains are indicated by the notation SUP[CtYFP1//Q47K], where the // separates the two pJSB constructs successively used to generate that strain.

Suppressor strains were apparently generated by random genomic mutations in *E*. *coli* cells expressing mutant or foreign FtsZ as their sole source of FtsZ; we did not use any mutagen. The relatively high frequency of the appearance of these suppressor strains (10^−4^ to 10^−6^) suggests that they were the result of a single nucleotide change in the genome, likely resulting in the loss-of-function of a gene product. Insertions and deletions may also occur at this frequency, and these may account for the two cases where we did not find a point mutation. Because *E*. *coli* has a spontaneous genome mutation frequency of 0.0025 per generation [23], we expected to find only a single, unique, nucleotide change in each of the 10 sequenced suppressor strains that were derived from a single FtsZ mutant. In the three cases of double suppressors, we expected to find two independent nucleotide changes in the genome, with each one accounting for the suppression of one of the two mutant FtsZ phenotypes.

### Genome alignment and detection of point mutations

We sequenced genomic DNA from 13 unique suppressor strains, generated from the expression of 7 different mutant or foreign *ftsZ* (Table 1). We obtained 23 to 42 million reads per strain, each with a length of 36 nucleotides, to cover the 4.62-Mb genome. We aligned these sequences to the published *E*. *coli* W3110 genome AP009048 [17] using the Bowtie sequence aligner package. Reads were aligned to the reference genome only if they contained two or fewer consecutive mismatched nucleotides, eliminating the necessity to screen mapped reads for poor quality or to trim reads of low-quality ends. This removed between 12% and 51% of reads from the final alignment. Further, reads were suppressed if they mapped to more than one location in the genome, which eliminated between 3% and 5% of all reads in each data set. The resultant final alignments provided an average coverage depth between 107X and 254X at each mapped nucleotide position (Table 2). We initially tried Samtools to identify SNPs between each strain and the reference genome. However, Samtools generated a number of false positives with <99% agreement of the reads; for unknown reasons Samtools also failed to reliably identify true SNPs (99–100% reads). We therefore used the Integrated Genome Viewer (IGV) program to visually scan each of the strain alignments. IGV provided visual identification of the mutation and a local alignment of all short reads contributing to the mutation call. All mutations identified were present in at least 99% of the reads aligned across the given nucleotide position, with the majority of the mutations occurring in 100% of the aligned short reads.

**Table 2:** Read statistics for sequence data and assembly of suppressor strain genomes. “Total Reads” indicates the number of 36-nt reads generated for each strain by Illumina GAII sequencing. “Aligned Reads” indicates the proportion of the total reads that were reliably aligned to the reference sequence. “Suppressed Reads” are reads that were excluded from the completed alignment because of regions of poor quality or because they aligned to more than one region of the genome. “Orphan Reads” are the proportion of the total reads that failed to align to the reference, most often due to very poor quality at the ends of the read. “Approximate Genome-Wide Depth of Aligned Coverage” is the average number of reads that aligned to each nucleotide in the genome, indicated the number of data points mapped for each nucleotide.

Strain	Total Reads	Aligned Reads		Suppressed Reads		Orphan Reads		Approximate Genome-Wide Depth of Aligned Coverage
SUP[Av3]	28891402	16404025	56.78%	1135072	3.93%	11352305	39.29%	131X
SUP[Av4]	25575027	13412300	52.44%	1101800	4.31%	11060927	43.25%	107X
SUP[Bs/EcCt1]	40737428	25464712	62.51%	1651984	4.06%	13620732	33.44%	204X
SUP[Bs/EcCt2]	37912312	16681192	44.00%	1848748	4.88%	19382372	51.12%	133X
SUP[D212G3]	23989718	20018951	83.45%	941445	3.92%	3029322	12.63%	160X
SUP[D212G9]	39865602	23804734	59.71%	1976229	4.96%	14084639	35.33%	190X
SUP[Mpu/EcCt13]	41766645	29344412	70.26%	1525937	3.65%	10896296	26.09%	237X
SUP[Mpu/EcCt28]	30542278	14687287	48.09%	1180475	3.87%	14674516	48.05%	117X
SUP[Q47K2]	24995916	18275930	73.12%	1000579	4.00%	5719407	22.88%	146X
SUP[CtYFP1//Q47K]	38136702	28493607	74.71%	1300019	3.41%	8343076	21.88%	228X
SUP[CtYFP1//D96A]	39987284	31740360	79.38%	1227588	3.07%	7019336	17.55%	254X
SUP[CtYFP1//D212G-YFP]	41485020	33118889	79.83%	1590292	3.83%	6775839	16.33%	332X
SUP[CtYFP2]	29613232	16716975	56.45%	1062132	3.59%	11834125	39.96%	134X

Independent alignment of each data set to the reference sequence gave individual genome files with very high coverage and quality. Reads that were discarded due to multiple mapping sites were fairly consistent between data sets and represented a low proportion of the total sequence data. These reads likely mapped to insertion elements within the *E*. *coli* genome, which are known to vary widely between strains.

In all sequenced suppressor strains we found 17 genomic mutations that differed from the W3110 sequence (Table 3). We concluded that these were differences between our parent JKD7 strain and the database W3110 strain. In 11 of the 13 sequenced strains we found one additional unique mutation (Table 4). We concluded that these were the suppressor mutation in that strain. In two strains (SUP[Bs/EcCt1] and SUP[CtYFP1//D212G-YFP]) we were unable to find any unique point mutation. Each mutation identified by genomic analysis was verified by direct sequencing of the relevant locus, obtained from PCR of the genomic DNA.

**Table 3:** Genomic mutations common to all sequenced suppressor strains. Base mutation locations in column 1 indicate mutated nucleotide numbering in the whole AP009048 reference genome. These mutations are identified as those where our JKD7-1 strain differs from the reference W3110 strain.

Base Mutations	Gene	Protein Function	Protein Mutation	b-num^a^	JW id^a^
A 547694 G	ylbE	hypothetical protein	E38E	b4572	JW0507
A 556858 T	folD	bifunctional 5,10-methylene-tetrahydrofolate dehydrogenase and 5,10-methylenetetrahydrofolate cyclohydrolase	L36Q	b0529	JW0518
C 663526 T	dacA	penicillin-binding protein 5	W286Stop	b0632	JW0627
T 825317 A	ybhQ	predicted inner membrane protein	L89Q	b0791	JW0774
G 987574 T	intergenic	-	-	-	-
T 1093686 C	ycdT	predicted diguanylate cyclase	V130A	b1025	JW5143
A 1103592 G	intergenic	-	-	-	-
C 1121926 T	solA	N-methyltryptophan oxidase, FAD-binding	E80K	b1059	JW1046
C 1303982 T	oppA	periplasmic-binding component of the ABC superfamily	Q363Stop	b1234	JW1235
A 1373590 T	ycjM	predicted glucosyltransferase	Q554L	b1309	JW1302
C 1966148 G	flhA	predicted flagellar export pore protein	R206P	b1879	JW1868
C 2005401 T	fliC	flagellar filament structural protein	E115K	b1923	JW1908
C 2045659 A	serU	tRNA-Ser		b1975	JWR0037
C 2778279 T	ypjA	adhesin-like autotransporter	S1035N	b2647	JW5422
A 2822193 G	recA	DNA strand exchange and recombination protein with protease and nuclease activity	L78P	b2699	JW2669
A 3266965 C	intergenic	-	-	-	-
C 3697802 A	rbsR	DNA-binding transcriptional repressor	G218V	b3753	JW3732

^a^The b number (b-num) and JW identifier (JW id) are the locus tags in the published W3110 genome (AP009048).

**Table 4:** Unique genomic mutations identified in sequenced suppressor strains. Base mutation locations indicate mutated nucleotide numbering in the whole AP009048 reference genome.

Strain	Base Mutations	Gene	Protein Function	Protein Mutation	b-num^a^	JW id^a^
SUP[Av3]	C 3899442 T	inter-genic	-	-	-	-
SUP[Av4]	G 989377 A	asnS	asparaginyl-tRNA synthetase	Q11Amber(S)	b0930	JW0913
SUP[Bs/EcCt1]	-	-	-	-	-	-
SUP[Bs/EcCt2]	TA 4608134–5 AT	inter-genic	-	-	-	-
SUP[D212G3]	G 2525323 A	gltX	glutamyl-tRNA synthetase	R265C	b2400	JW2395
SUP[D212G9]	T 439585 G	ispA	geranyltranstransferase	K247N	b0421	JW0411
SUP[Mpu/EcCt13]	T 2767761 A	yfjQ	prophage CP4-57	S147R	b2633	JW2614
SUP[Mpu/EcCt28]	A 433159 C	ribD	fused diaminohydroxyphosphoribosylaminopyrimidine deaminase and 5-amino-6-(5-phosphoribosylamino uracil reductase)	T161P	b0414	JW0404
SUP[Q47K2]	G 3308772 A	nlpI	conserved hypothetical protein	P3V	b3163	JW3132
SUP[CtYFP1//Q47K]	C 1738186 A	ydhP	predicted transporter	L273P	b1657	JW1649
SUP[CtYFP1//D96A]	G 2438847 A	accD	acetylCoA carboxylase, beta (carboxyltransferase) subunit	Q176Amber(S)	b2316	JW2313
SUP[CtYFP1//D212G-YFP]	-	-	-	-	-	-
SUP[CtYFP2]	G 3746911 A	tnaB	low-affinity tryptophan transporter	P24S	b3709	JW5619 JW5622

^a^The b number (b-num) and JW identifier (JW id) are the locus tags in the published W3110 genome (AP009048).

Since we sequenced 13 suppressor strains we did not need to sequence our JKD7-1 parental lab strain. The 17 mutations common to all suppressor strains were classified as parental strain mutations of JKD7-1 relative to the published W3110 reference genome. These mutations are all in non-essential genes or non-coding regions of the DNA, or were point mutations that apparently did not eliminate function. This number of differences between the lab strain and published genome is consistent with, but somewhat larger than, previously reported re-sequencing projects of laboratory bacteria strains [24, 25].

One parental mutation was identified as a key difference between the JKD7-1 lab strain and the published W3110 genome: the C to A transition at genome position 2045659 occurs in the anti-codon of a Ser-tRNA. This mutation alters the anti-codon of the Ser-tRNA from CGA to CTA, which allows that Ser-tRNA to recognize and bind the TAG amber stop codon. With this mutation, our lab strain is an amber suppressor, occasionally misincorporating serine into growing peptides at the amber stop codon. Two of our suppressor mutations are in essential genes, and depend on the amber suppression: a CAG (Gln) to TAG (amber stop) at residue 11 of asnS (asparaginyl-tRNA synthetase), and a similar mutation at residue 176 of accD (acetylCoA carboxylase beta). The amber suppression results in a Gln to Ser substitution, which might also affect the amount of translated protein.

A single, unique nucleotide mutation was identified in 11 of the sequenced suppressor strains. The mutations found were different in each strain, even when two strains were derived from the same initial non-functional FtsZ, e.g., SUP[Av3] and SUP[Av4]. Two of the mapped SNPs were in intergenic regions that do not correspond to a coded protein. The remaining nine mutations all change one amino acid in a protein. The mapped mutations occurred in five essential and four non-essential *E*. *coli* genes, and corresponded to an amino acid change or a premature stop codon in the identified genes. None of the identified mutated proteins are known to play a role in bacterial cell division. Most genes appear to be involved in secondary metabolism, or pathways for synthesis of biological macromolecules, thus affecting cell growth and general health.

### Complementation in slow growth conditions

We hypothesized that the function of some of the suppressor mutations might be based on a general decrease in cell health, perhaps weakening the cell wall or slowing the cell cycle, to facilitate division by the weakened FtsZ. A precedent is the observation that cells with a double deletion of SlmA and Min could grow on M9 minimal medium, but not on LB [26]. We examined the ability of the mutant or foreign FtsZ to complement an *ftsZ*-null phenotype when cell growth was slowed.

We tested the seven FtsZ mutant constructs that generated the suppressor strains examined here. Additionally, we tested pJSB-Pa, which expresses the *P*. *aeruginosa* FtsZ and generates a suppressor strain that was not sequenced in this study. pJSB2 constructs were freshly transformed into JKD7-1/pKD3 cells. The cells were grown to stationary phase in LB-Repression Media, then diluted to plate ~10^6^ cells on Induction Minimal Media.

Results are listed in Table 5. We found that the constructs expressing foreign FtsZ from *A*. *vinelandii* or *P*. *aeruginosa* were able to complement when grown in minimal medium, whereas in rich medium they needed to generate suppressor strains [14]. The other seven mutant FtsZs did not grow in minimal medium. Moreover, although we plated enough cells to allow for the generation of a suppressor strain, we did not observe any colonies. Slower growth is therefore not a general cure for weakened FtsZ mutants, and in most cases seems to inhibit the generation of suppressor strains.

**Table 5:** Complementation results in slow growth conditions. Column 1 lists each of the EcFtsZ mutants or foreign FtsZ tested. Column 2 lists the suppressor strains that were sequenced in the present study. In column 3 ++ means ~>80% of the cells plated formed a colony on the M9 plate; –means that colony formation was < 10^−5^, i.e. there was no complementation and suppressor strains did not arise.

Plasmid	Resultant Suppressor Strain(s)	Complementation in Minimal Media
pJSB2-Av	SUP[Av3], SUP[Az4]	++
pJSB2-Bs/EcCt	SUP[Bs/EcCt1], SUP[Bs/EcCt2]	-
pJSB2-D212G	SUP[D212G3], SUP[D212G9]	-
pJSB2-D212G/CtYFP	SUP[CtYFP1//D212G-YFP]	-
pJSB2-Mpu/EcCt	SUP[Mpu/EcCt13], SUP[Mpu/EcCt28]	-
pJSB2-Q47K	SUP[Q47K2], SUP[CtYFP1//Q47K2]	-
pJSB2-D96A	SUP[CtYFP1//D96A]	-
pJSB2-CtYFP	SUP[CtYFP1//Q47K], SUP[CtYFP1//D96A], SUP[CtYFP1//D212G-YFP], SUP[CtYFP2]	-
pJSB-Pa	None reported here	++

### Intragenic suppressors

We chose three mutants (R174A, E250K and L272V), which did not complement or generate genomic suppressor strains, to screen for second-site, intragenic suppressor mutations. R174A was originally suggested to block assembly of protofilament bundles in Ca^2+^ [27], perhaps by blocking lateral interactions, but our lab has found that bundle formation is normal [28]. Nevertheless it is non-complementing and apparently toxic, since we are unable to maintain a freezer stock of a pET plasmid in BL21. E250K was initially examined as a deleterious double mutant, E250K/D253K [19, 29]. Individually, we found that E250K was the most severe of these two mutations, abrogating FtsZ function in vivo on its own. L272 is a very sensitive amino acid for which only two substitutions complement: L272M/I [30]. We chose the conservative substitution L272V, which is nevertheless non-functional. We created pJSB2 plasmids with these three non-functional mutants, and generated a library of FtsZ with additional random mutations, aiming for less than one additional mutation per FtsZ.

The screen of R174A produced two suppressors, L169V and L169Pthat were verified as able to complement R174A. L169 is a well conserved residue, and in the FtsZ structure, is solvent exposed on the upper right face of the subunit, very near R174. The related mutant L169R has been found to reduce sensitivity to FtsZ inhibitors Kil, MinCD and SulA and to bypass the need for ZipA, perhaps by enhancing protofilament bundling [31, 32].

The screen of L272V produced three second site mutations that appeared to suppress the non-functional L272V phenotype: E305V, V311A, and R202C. All three of these mutations were able to function for cell division, either alone or as a double mutant with L272V. However, E305V and R202C required a genomic suppressor mutation when expressed alone. E305V and V311A were isolated together in the screen, as an L272V/E305V/V311A triple mutant, and both the E305V/V311A double mutant and the triple mutant were functional. We have not tested E305V and V311A as single mutants.

The screen of E250K produced one second site mutation that appeared to suppress the non-functional E250K phenotype: D253E. This conservative mutation elongates the side chain by one CH_2_, which may allow this negatively charged side chain to form a salt bridge with the 250K, neutralizing the positive charge that rendered the original mutant non-functional.

The intragenic suppressors are summarized in Table 6.

**Table 6:** Intragenic suppressors of three non-functional FtsZ point mutants. ++ indicates that the mutant complemented in the JKD7/pKD3 system. (+) indicates that it did not complement but could generate suppressor strains.

Initial Mutation for Screen	Identified Second Site Mutation	Complementation: Second Site Mutation Alone	Complementation: Initial and Second Site Mutations Together
R174A	L169V	++	++
	L169P	++	++
L272V	E305V	++	(+)
	V311A	++	++
	R202C	++	(+)
	E305V / V311A	++	(+)
E250K	D253E	++	++

## Discussion

Whole genome re-sequencing has been shown to be a reliable method for identifying single bp mutations in the genome of bacteria [15–17, 24, 25]. Here, we have shown that each of our suppressor strains differs from the most recently published W3110 genome at 17 locations, which we interpret to be changes in our parental lab strain. Other studies that have compared newly sequenced *E*. *coli* genomes to the published reference sequence have found a conservative number of changes. While we found 17 parental mutations, Skovgaard et al [25] found seven parental mutations between their suppressor strains and the published MG1655 sequence [17]. One of those mutations (A 547694 G) also occurred in our lab strain, a W3110 strain.

We identified a single suppressor mutation in 11 of the 13 sequenced strains, but no unique mutations were found in SUP[Bs/EcCt1] and SUP[CtYFP1//D212G-YFP]. We also were only able to find one mutation in SUP[CtYFP1//Q47K] and SUP[CtYFP1//D96A]. Those strains and SUP[CtYFP1//D212G-YFP] were double suppressors, and should therefore have two independent mutations. Because the double suppressors were all initially generated as a single suppressor strain against the CtYFP FtsZ, we conclude that we did not identify the CtYFP suppressing mutation. The two mutations that we did find likely correspond to the second mutation, generated by Q47K or D96A. This indicates that there were three mutations that we did not find in our sequenced genomes. Given the nature of Illumina whole genome sequencing, which produces millions of short reads (36 nucleotide each) that can be aligned to a total genome, it is often impossible to identify large insertions, deletions or chromosomal rearrangements between a sequenced genome and the reference. It seems likely that the three missing mutations were due to insertions or deletions that our analysis could not detect. Skovgaard et al [25] showed that copy-number analysis could detect larger chromosomal rearrangements. We did not attempt this extra step.

Most of the identified suppressor mutations are not immediately obvious for playing a role in bacterial cell division. We did, however, find mutations in three genes, *ispA*, *accD* and *nlpI*, that may offer interesting new lines of inquiry, as indicated by independent discoveries of these genes affecting cell division.

*ispA* is an essential gene encoding a farnesyl diphosphate synthase [33], an enzyme involved in farnesyl pyrophosphate production and belonging to the pathway producing bactoprenol for peptidoglycan synthesis. The SUP[D212G9] mutation (T 2767761 A) encodes a lysine to asparagine substitution at position 247 of IspA. This removes a positive charge very near one of the two “aspartate-rich domains” believed to be important for catalysis [34, 35]. This mutation may compromise isoprenoid synthesis in the suppressor strain, leading to a loss of integrity of the peptidoglycan layer, which could allow for a mutant FtsZ producing a lower constriction force to invaginate the membrane and initiate cell division. Leaver et al [24] found that a mutation in the *B*. *subtilis yqiD* gene, which is a homolog of *E*. *coli ispA*, was essential to facilitate isolation of L forms, which can grow and divide without a cell wall. Kawai et al [36] later showed that this was due to reduction in reactive oxygen species generated by the electron transport chain. Shiomi and Niki found that a mutant of *E*. *coli ispA* suppressed the low-temperature lethality of a *RodZ* deletion [37].

*accD* encodes one subunit of acetyl-CoA carboxylase, which carries out the first committed step of fatty acid synthesis. *accD* is considered an essential gene [38]. The Q176S mutation may result in a partial loss of function. In a recent study of *B*. *subtilis* L forms, it was found that overexpression of *accDA* (the operon *accD-accA* was expressed together) could support L-form growth in much the same way as inhibition of proteoglycan precursor synthesis [39]. The overexpression was lethal in the wild type state, with cell walls. It is intriguing that mutations in *accDA* have appeared in both the L-form study and as one of our FtsZ suppressor strains.

*nlpI* is a membrane-associated lipoprotein with an unclear function. However, perturbation in NlpI levels in *E*. *coli* causes dramatic changes to cell morphology, including filamentation in *nlpI*-null cells and the formation of single prolate ellipsoids in cell overexpressing NlpI [40]. The SUP[Q47K2] mutation (G 3308772 A) encodes a proline to valine substitution at position 3 of NlpI. This is at the start of the protein’s signal sequence, and may affect protein expression levels. The ability of a P3V mutation in NlpI to allow Q47K FtsZ to function for cell division, taken together with the previously demonstrated septation defects of *nlpI*-null cells suggest that *nlpI* may be involved in the division process, perhaps at the level of remodeling the cell wall.

Apart from *ispA*, *accD* and *nlpI*, the suppressor mutations we found for mutant *ftsZ* were mostly in metabolic pathways with no obvious or direct link to cell division. This contrasts with a recent study of suppressors of the cell shape gene *rodZ* [41]. There, suppressors were found in *rodA* and *mreB*, whose proteins interact directly with RodZ. A difference between the two cases is probably in the frequency at which the suppressor mutations arose. In the RodZ study the frequency is not known, but cells were selected over five to seven days of growth in liquid culture, suggesting that they may be quite rare. This would be expected if suppressors could only be obtained in two specific genes. In our case mutations arose at relatively high frequency: 10^−4^ to 10^−6^ of the cells plated developed into a colony upon overnight culture on a plate. We previously suggested [14] that suppressors of *ftsZ* might be obtained by mutations in multiple genes in multiple pathways. In the present study we sequenced two independent suppressor strains generated by Av, Bs and D212. Each suppressor strain had a single mutation in a different gene, consistent with the suggestion of multiple genes in multiple pathways.

The search for intragenic suppressors focused on aa’s that were near the longitudinal protofilament interface (R174A and L272V) and on the side (E250K). We had hoped to find intragenic suppressors on the opposite protofilament interface or perhaps on the opposite side to identify a lateral bond. However the suppressor mutations were all reasonably close to the original mutation, suggesting that they caused minor changes in the structure of the sub-domain that restored activity. We note that the mutagenesis was probably not saturating, so additional mutations may have been missed.

When suppressor mutations are identified by genome sequencing, it is generally important to confirm them by introducing them into the genome of the parent strain and demonstrating that this re-creates the suppressor phenotype. Readers should be aware that none of our mutants have been confirmed by the gold standard of re-introducing them in the mother strain. This is certainly important if one is characterizing a single suppressor strain. In our study, however, we have characterized multiple strains, which provide internal controls that obviate the need for this reconstitution. First, the 17 SNPs we found relative to the database W3110 strain were all found in each of the 13 strains that we sequenced. This demonstrates the reproducibility of the sequencing. Second, the 11 SNPs we identified as suppressor mutations were each unique to that one suppressor strain, and were found in 99–100% of reads for that strain. Third, the coincidence of finding an amber suppressor mutation in our parent strain, and two amber-stop mutations in essential genes, demonstrate internal consistency. Finally, we emphasize that we are not making strong claims for any of the suppressor mutations. At the present time they are simply suggestions of pathways that might be followed up in the future. Our general conclusion is simply that multiple metabolic pathways can feed back to cell division, a conclusion that has already been determined for specific pathways [42, 43].

Hill et al [42] identified one pathway by which metabolism feeds back to modulate FtsZ and cell division. They found that UDP-glucose serves as an intracellular proxy for nutrient availability, and operates through the glucosyltransferase Ought to regulate cell division. In nutrient-rich conditions, abundant UDP-glucose activates OpgH to bind and sequester FtsZ, inhibiting cell division and increasing cell size. In nutrient-poor conditions UDP-glucose is decreased and OpgH is inactivated, releasing FtsZ for more active cell division. Any metabolic pathway affecting the level of UDP-glucose could thereby affect cell division. Some of the suppressor mutations we have identified may be operating through this OpgH regulation. Others may be operating through additional pathways. See [43] for a recent review of metabolic pathways moderating bacterial division.

Our conclusion that alterations in several pathways of general cell metabolism can feed back and modulate cell division is similar to findings in a recent study of *B*. *subtilis* L forms. In the original study, Leaver et al [24] found that two alterations were required to generate stable L forms: inhibition of cell wall synthesis through depletion of MurE, plus a mutation in *ispA*. In later study, Mercier et al [39] reported that “the *ispA* mutation can be substituted by mutations in many genes on different metabolic pathways.” The multiple metabolic pathways that can provide our suppressor mutations are likely related to those involved in L form stability. However the feedback of these pathways to cell division is likely complex and peculiar to each pathway.
